# The association of the quality of sleep with proinflammatory cytokine profile in inflammatory bowel disease patients

**DOI:** 10.1007/s43440-021-00333-0

**Published:** 2021-10-25

**Authors:** Aleksandra Sobolewska-Włodarczyk, Marcin Włodarczyk, Marcin Talar, Maria Wiśniewska-Jarosińska, Anita Gąsiorowska, Jakub Fichna

**Affiliations:** 1 Department of Biochemistry, Medical University of Lodz, 92-215 Lodz, Lodz, Poland; 2 Department of Gastroenterology, Medical University of Lodz, Lodz, Poland; 3 Department of General and Oncological Surgery, Medical University of Lodz, Lodz, Poland

**Keywords:** Circadian rhythm abnormalities, Quality of sleep, Inflammatory bowel diseases, Crohn’s disease, Ulcerative colitis

## Abstract

**Background:**

The role of circadian rhythm abnormalities in patients with inflammatory bowel disease (IBD) remains relatively unknown. The aim of this study was to identify the inflammatory cytokine profile in the IBD patients and its relationship with the quality of sleep.

**Methods:**

Prospective, single-center observational cohort study was performed. In all enrolled adult IBD patients, the disease activity was assessed using Crohn’s Disease Activity Index (CDAI) for Crohn’s disease (CD) and Partial Mayo Score for ulcerative colitis (UC), respectively. To assess the quality of sleep, all patients were asked to respond to a questionnaire to define Pittsburgh Quality Sleep Index (PSQI). From all enrolled patients, 15 ml venous blood was taken to determine serum inflammatory cytokine levels and perform standard laboratory tests.

**Results:**

Fifty-two IBD patients were enrolled in the study: 32 with CD and 20 with UC. The poor sleep was noted in 69.4% of patients with clinically active and in 6.3% of patients with inactive disease. In the group of IBD patients with poor sleep, the significantly higher level of serum IL-6, IL-17, and IL-23 were observed. In IBD patients with exacerbation, the significantly higher level of serum IL-6, IL-17, and IL-23 were recorded.

**Conclusions:**

The relationship between quality of sleep and proinflammatory cytokine profile may show us a predisposition for the development of inflammatory intestinal lesions in IBD patients with sleep disturbances. This knowledge may allow the pharmacological and behavioral therapies of circadian rhythm abnormalities to become new significant targets in IBD patients.

## Introduction

Inflammatory bowel disease (IBD), including two main representatives Crohn’s disease (CD) and ulcerative colitis (UC), are chronic health problems of difficult management and treatment. Despite numerous recent studies, currently the IBD pathogenesis has not been fully determined. It have been shown a similar proinflammatory cytokine profile as in rheumatoid arthritis, psoriasis, and other immunological pathologies [1–3]. Nevertheless, genetic predisposition together with the environmental and infectious factors, lead to exacerbation of inflammatory lesions in IBD patients [4, 5].

Over 75% of patients with active CD and UC suffer from self-reported poor sleep [7, 8]. Sleep abnormalities among IBD patients predict increased risk of surgery and hospitalization [9] and are independently associated with reduced quality of life [8, 10]. The two-way relationship of IBD with sleep disorders is due to the fact that exacerbation of the disease leads to sleep disorders, which in turn increase the activity of the disease [7, 8]. Circadian rhythm abnormalities may significantly impair the gut-brain axis, what may lead to changes in cortisol levels that reach a nadir at the beginning of sleep and a peak before awakening [9]. In addition, sleep may strengthen long-term memories in the immune system and increase the activity of the complement system [10, 11]. The adverse effects of chronic sleep deprivation comprise an enhanced risk for various diseases as a consequence of a persistent low-grade systemic inflammation on the one hand, as well as a manifest immunodeficiency characterized by an enhanced susceptibility to infections and a reduced immune response to vaccination on the other hand [12]. However, the inflammation may also significantly affect the quality of sleep. For instance, it was proven that the administration of interleukin-1-β (IL-1-β), tumor necrosis factor (TNF), or interferon-α (IFN-α) into the cerebral ventricle of rabbits enhances the slow-wave sleep [13]. In the other animal study, the increase of IL-1β, IL-6 and TNF-α levels were associated with non-rapid eye movement (NREM) sleep [6]. In our previous observational study, a strong relationship between clinically active IBD and sleep disturbances was observed [7, 8]. This finding may indicate circadian rhythm disorders as a risk factor of exacerbation of IBD. Overall, there are too few reports to confirm that sleep disturbances are a risk factor of IBD.

In the current study, we sought to characterize specific inflammatory cytokine profile among individuals with CD and UC and investigate the overlap between the quality of sleep and disease course.

## Methods

### Ethical considerations

All approvals required to perform this study were obtained from the Committee of Bioethics of Medical University of Lodz (RNN/621/14/KB). The study enrolled only adult patients with the confirmed diagnosis of IBD who gave their written and informed consent to participate.

### Patients

This prospective clinical cohort study was performed in 52 adult patients (26 men and 26 women; with mean age ± SD (standard deviation) 39.7 ± 15.3 years), who were hospitalized from January 2017 to December 2019 at the Department of Gastroenterology at the Medical University of Lodz, Poland. Adult individuals with confirmed diagnosis of IBD were enrolled (CD n = 32, UC n = 20). The case report form for each patient qualified for the study including age, gender, body mass index, comorbidities, and smoking history were collected. Current smokers, patients with a history of cardiovascular disease, pulmonary and kidney disease, allergy, diabetes, lichen planus, psoriasis, atopic dermatitis and other autoimmune skin lesions and those treated with anti-inflammatory drugs (except azathioprine), antioxidants, or statins were excluded from the study.

### Assessment of disease activity

In all IBD individuals, the clinical state disease was assessed using validated scales, including Crohn’s Disease Activity Index (CDAI) for CD and Partial Mayo Score for UC. According to European Crohn’s and Colitis Organization (ECCO) guidelines presented in *The second European evidence-based consensus on the diagnosis and management of Crohn's disease*, for CD, CDAI < 150 was defined as remission; CDAI 150–220 with no features of obstruction, fever, dehydration, abdominal mass, or tenderness was defined as mild CD; CDAI 220–450 or intermittent vomiting, or weight loss > 10% or treatment for mild disease ineffective, or tender mass with no overt obstruction was defined as moderate CD; CDAI < 450 or cachexia (BMI < 18 kg m − 2), or evidence of obstruction or abscess or persistent symptoms despite intensive treatment was defined as severe CD. For UC according to ECCO’s *Third European evidence-based consensus on the diagnosis and management of ulcerative colitis*, clinical remission was defined as a Mayo Score of 0; mild UC was defined as Mayo Score of 1; moderate UC was defined as Mayo Score of 2; severe UC was defined as Mayo Score of 3.

### Assessment of circadian rhythm abnormalities

All patients were asked to respond to a questionnaire to define Pittsburgh Quality Sleep Index (PSQI). PSQI was introduced in 1989 by Buysse et al. to standardize and objectify the circadian rhythm abnormalities [11]. PSQI is a widely used sleep evaluation tool, which reflects the sleep abnormalities through seven components and considers recalling events in the past month. This questionnaire includes nineteen individual items, which concur to generate seven components referring to subjective sleep quality, use of sleeping medication, sleep latency and duration, habitual sleep efficiency, daytime dysfunction. Patients can evaluate each component and the result can range from “0” in cases of no impairment to “3” in cases of severe difficulty. All scores are combined according to the scoring criteria included with the form to produce a Global PSQI Score. Scores above 5 indicate clinically meaningfully disturbed or poor sleep.

The PSQI form used in the study was a translated version of the original one (the applicant has obtained consent from the authors of the original survey for use in the study). The original English version was translated into Polish and consulted with a psychologist specializing in IBD.

### Collection of tissue and blood samples

In each subject enrolled to the study, 15 ml of venous blood was collected to serum vacuum tubes. Samples were left to clot for 30 min at room temperature before centrifugation at 1500×*g* for 10 min. Next, serum was collected and was stored at  − 80 °C for further biochemical analysis. Additional laboratory tests, including peripheral complete blood count and serum C-reactive protein (CRP) using automatic devices, were also performed at same time in each patient.

### Cytokine serum levels quantification

The serum concentrations of human IL-6, IL-17, and IL-23 were determined by the quantitative sandwich enzyme-linked immunosorbent assay (ELISA), using kits from Elabscience, USA. All tests were performed according to manufacturer’s instructions. Absorbance was read in a microplate reader at 450 nm (VICTOR X4, Perkin Elmer, USA). Each determination was carried out in triplicate in accordance with the principles of laboratory. Quantitative determination of interleukins in the samples was calculated by comparing optical density of the samples to the standard four parameter logistic curves.

### Statistical analysis

The data were analyzed using the Statistica 13.1 (StatSoft, Inc., United States). A Shapiro–Wilk test was used to determined normality of distribution; continuous variables were expressed as mean ± SD, demographic categorical data were described with absolute frequencies and percentages. Receiver operating characteristic (ROC) curves were constructed for the PSQI analysis, and the areas under the ROC curves with 95% confidence intervals (CIs) were calculated and compared with each other. Optimal cutoff values for PSQI, used to discriminate patients with clinical exacerbation of disease, were calculated by ROC curves. Sensitivity, specificity, and positive and negative predictive values (PPV, NPV, respectively) of the cutoff values and association were analyzed. The statistical significance between the groups was determined using unpaired *t* test or two-way ANOVA followed by followed the Tukey’s post hoc test. Correlations between variables were evaluated using the Pearson’s test or Spearman’s rank correlation coefficient test depending on normality. A value of * p* < 0.05 was considered statistically significant.

## Results

### Baseline characteristics

Fifty-two patients were enrolled in our study: 32 with CD and 20 with UC. The analysis of intergroup differences in the baseline characteristics of individuals enrolled are presented in Table 1. The homogeneity of analyzed groups was confirmed in terms of gender, age, body mass index (BMI), inflammatory markers, disease characteristics, and PSQI score.

**Table 1:** Baseline characteristics data, laboratory findings, and treatment history for patients enrolled in the study

	Crohn’s disease	Ulcerative colitis	*p* value
Subjects, *n* (%)	32 (62%)	20 (38%)	NA
Sex			0.776
Women, *n* (%)	16 (31%)	10 (19%)	
Men, *n* (%)	16 (31%)	10 (19%)	
Age, y	38.7 ± 12.5	41.5 ± 19.1	0.535
BMI, kg/m^2^	20.5 ± 1.8	21.4 ± 2.5	0.789
Disease duration (years)	7.4 ± 9.2	5.4 ± 5.6	0.469
Disease activity			0.183
Active, *n* (%)	20 (63%)	16 (80%)	
Remission, *n* (%)	12 (37%)	4 (20%)	
Steroids use, *n* (%)	13 (41%)	10 (50%)	0.508
CRP, mg/l	37.3 ± 61.9	29.7 ± 40.5	0.643
PSQI score	6.3 ± 2.8	6.1 ± 3.4	0.763
PSQI > 5, *n*	17	9	0.569

Data are presented as mean ± standard deviation (SD) or percentage as appropriate. Comparisons between groups were performed using the Student’s t-test and χ2 test

*BMI* body mass index, *CRP* C-reactive protein, *PSQI* Pittsburgh Quality Sleep Index, *NA* not applicable

### Analysis of circadian rhythm abnormalities among IBD patients

**Fig. 1:**
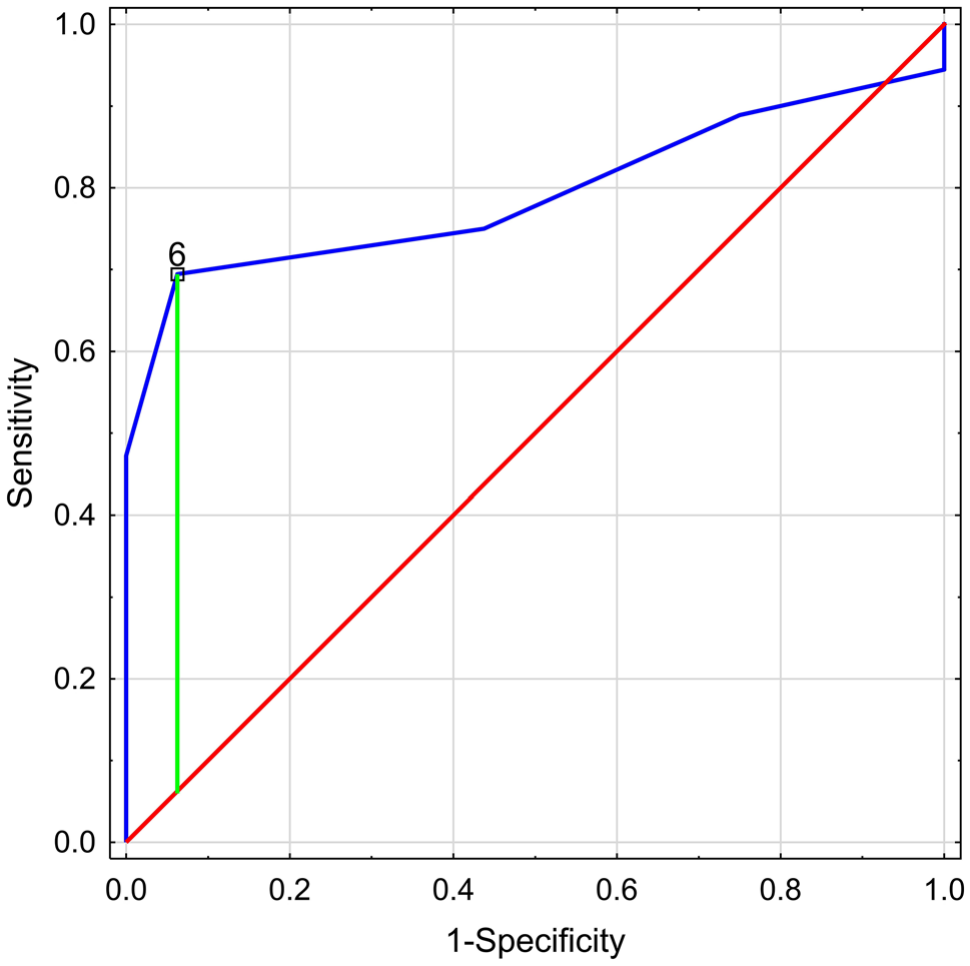
Receiver operating characteristic (ROC) curves for PSQI score and the clinical exacerbation of disease in Crohn’s disease and ulcerative colitis patients (AUC: 0.793; 69% sensitivity and 94% specificity)

**Fig. 2:**
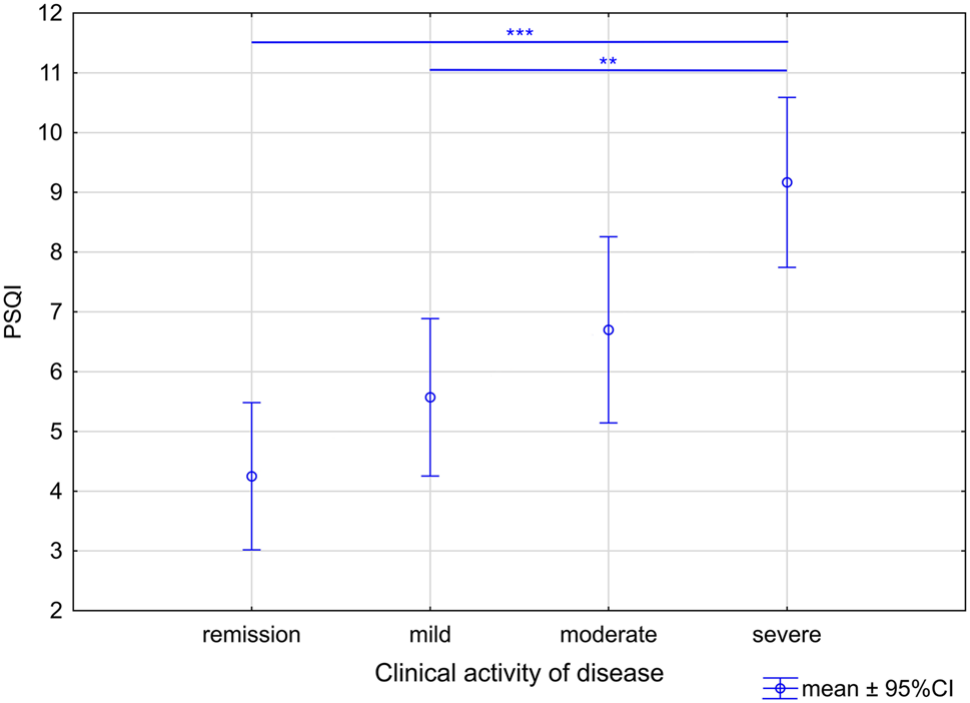
The relationships between Pittsburgh Quality Sleep Index (PSQI) score and clinical status of disease in inflammatory bowel diseases patients (*F*3,48 = 9.69; *p* < 0.001). Data are expressed as mean ± 95% CI. Statistical significance from the one-way ANOVA followed by Tukey’s post hoc test. Post hoc significant differences: PSQI: remission vs. severe (*p* < 0.001); mild vs. severe (*p* = 0.003)

The poor sleep was noted in 50.0% (26/52) of IBD patients enrolled to our study: in 69.4% (25/36) patients with clinically active and in 6.3% (1/16) patients with inactive disease (*p* = 0.001; OR 34.1, 95% confidence interval, 3.9–291.2). A global PSQI score of 5 points yielded a sensitivity of 75%, a specificity of 56%, and a positive predictive value of 79% for discriminating participants with exacerbation of IBD from those in clinical remission. In the ROC curves analysis of optimal cutoff values associated with clinical exacerbation of disease, the area under curve was 0.793 for PSQI score. The Youden analysis showed that PSQI higher than 6 indicates the exacerbation of IBD with 69% sensitivity and 94% specificity (Fig. 1). In unpaired t test, there was no relationship between PSQI score and subtype of IBD (CD vs UC; 6.3 ± 2.8 vs. 6.1 ± 3.4; *p* = 0.763; *t * = 0.303; df = 50). However, in one-way ANOVA, we noted significant differences in sleep abnormalities related to clinical state of the disease, and as revealed by the Tukey’s post hoc test the poorest sleep quality was reported in IBD patients with severe exacerbations (*F*_3,48_ = 9.69; *p* < 0.001) (Fig. 2). No statistical relationship between CRP level and PSQI score was detected (*r* = 0.155; *p* = 0.303). Further, no statistically significant correlation between disease duration and PSQI score was observed (Pearson’s test; * r* = 0.177; *p* = 0.342). Furthermore, there were no relationships between applied steroids in IBD patients and observed sleep abnormalities related with PSQI score above 5 points (*χ*2 = 1.94; *p* = 0.163).

### Analysis of relationship between cytokine profile and circadian rhythm abnormalities among IBD patients

In our analysis, there were no differences between CD and UC patients in levels of serum IL-6, as assessed by unpaired * t* tests (18.53 ± 16.72 pg/mL vs. 29.23 ± 22.66 pg/mL; *p* = 0.217; * t* =  − 1.274; df = 20), IL-17 (155.21 ± 129.69 pg/mL vs. 205.99 ± 136.18 pg/mL; *p* = 0.382; * t*  =  − 0.382; df = 20), and IL-23 (140.09 ± 104.59 pg/mL vs. 137.07 ± 139.28 pg/mL; *p* = 0.954; * t*  = 0.058; df = 20).

**Fig. 3:**
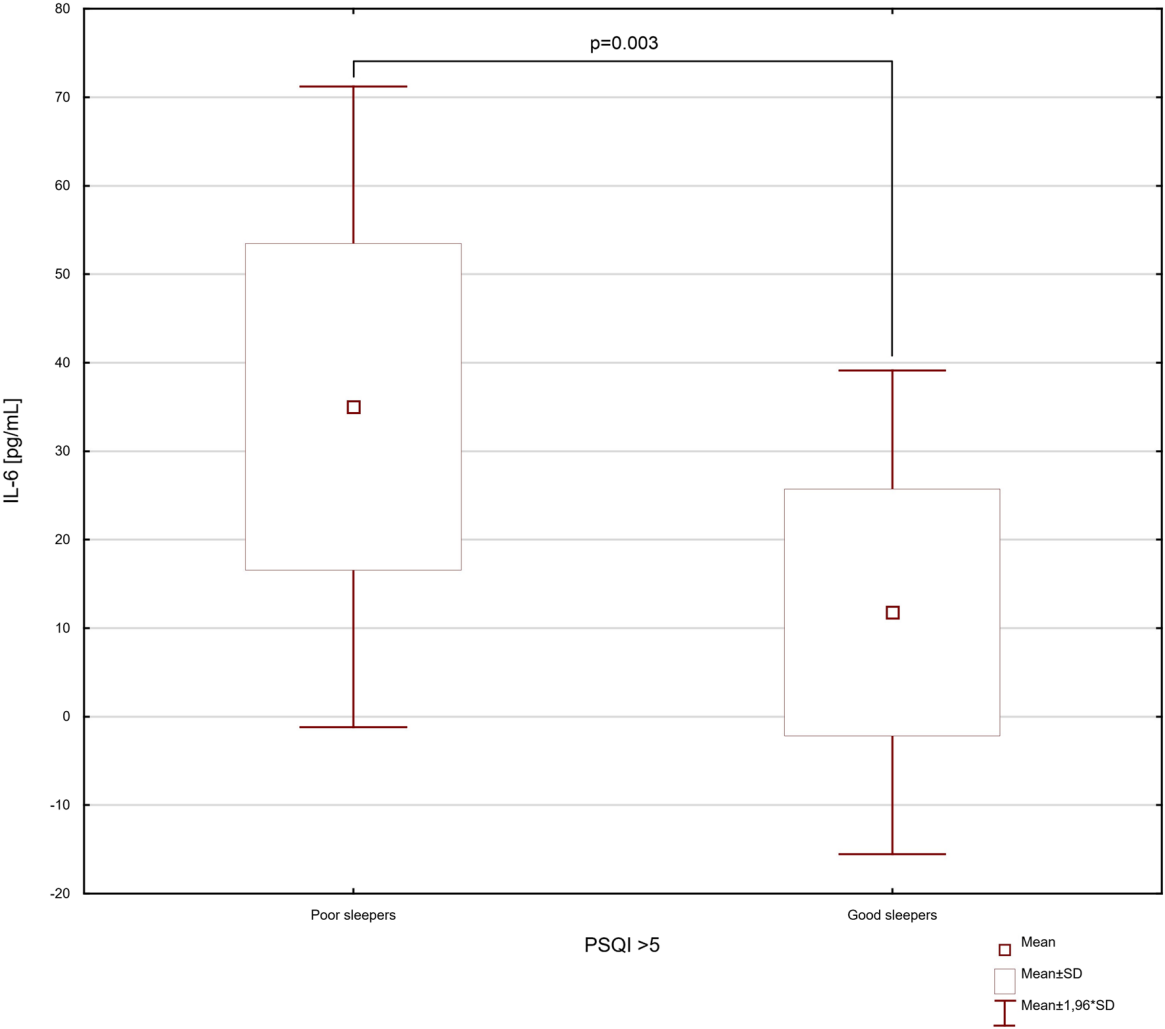
The difference in serum level of IL-6 in inflammatory bowel disease patients discriminated into two groups according to their sleep quality. Data are expressed as mean ± SD and mean ± 1.96*SD. Statistical significance was determined using unpaired *t* test

**Fig. 4:**
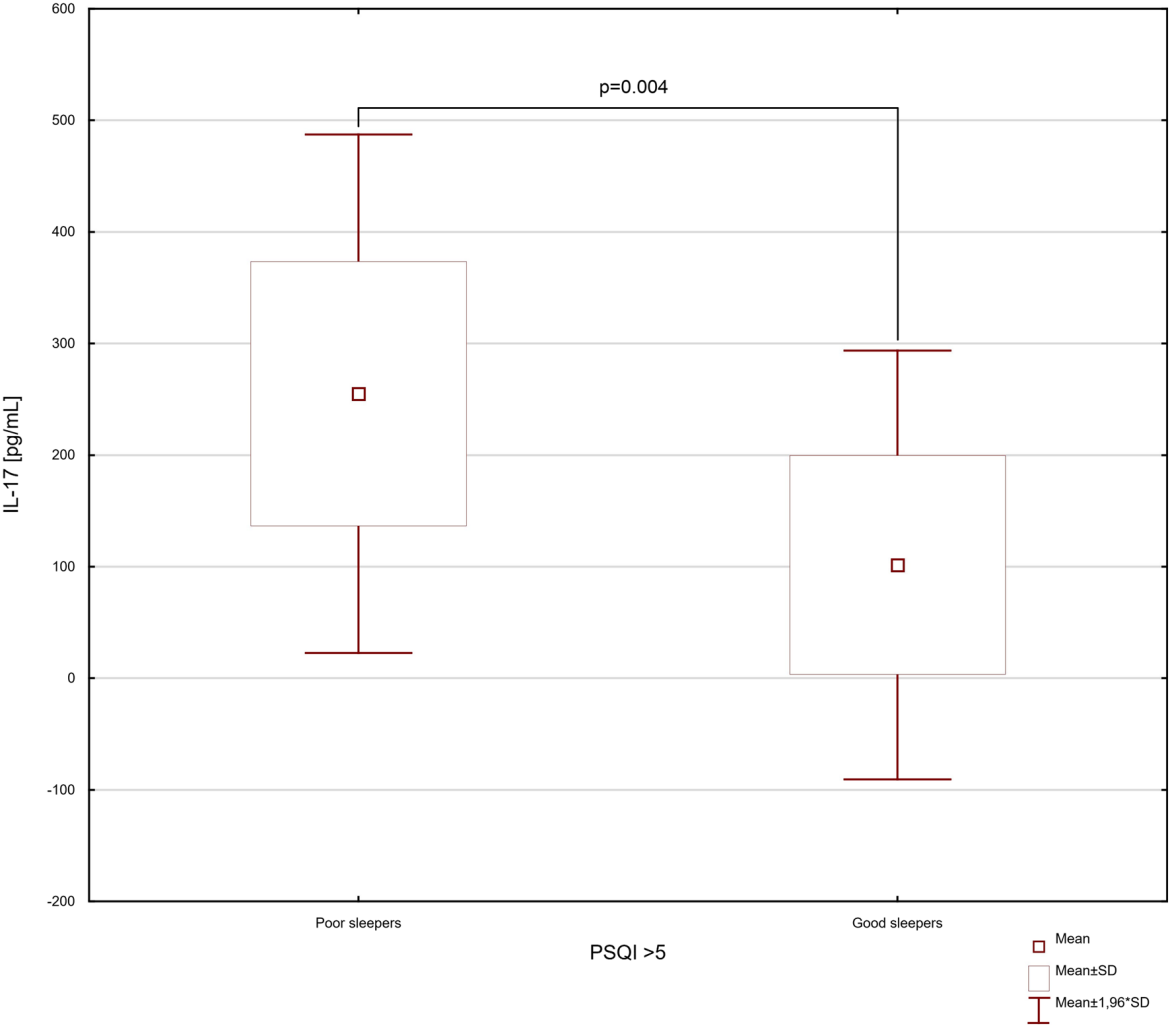
The difference in serum level of IL-17 in inflammatory bowel disease patients discriminated into two groups according to their sleep quality. Data are expressed as mean ± SD and mean ± 1.96*SD. Statistical significance was determined using unpaired *t* test

**Fig. 5:**
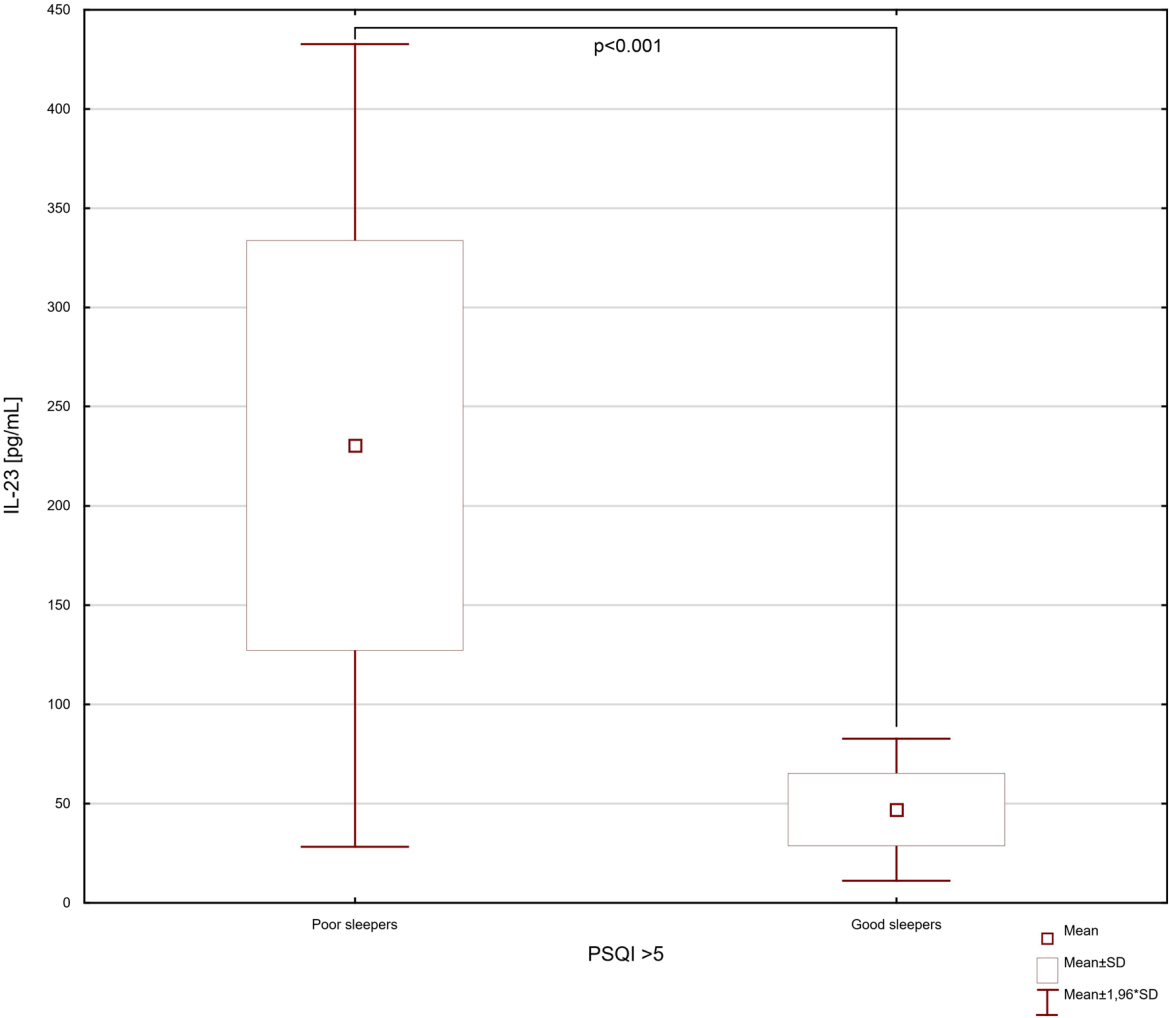
The difference in serum level of IL-23 in inflammatory bowel disease patients discriminated into two groups according to their sleep quality. Data are expressed as mean ± SD and mean ± 1.96*SD. Statistical significance was determined using unpaired *t* test

**Fig. 6:**
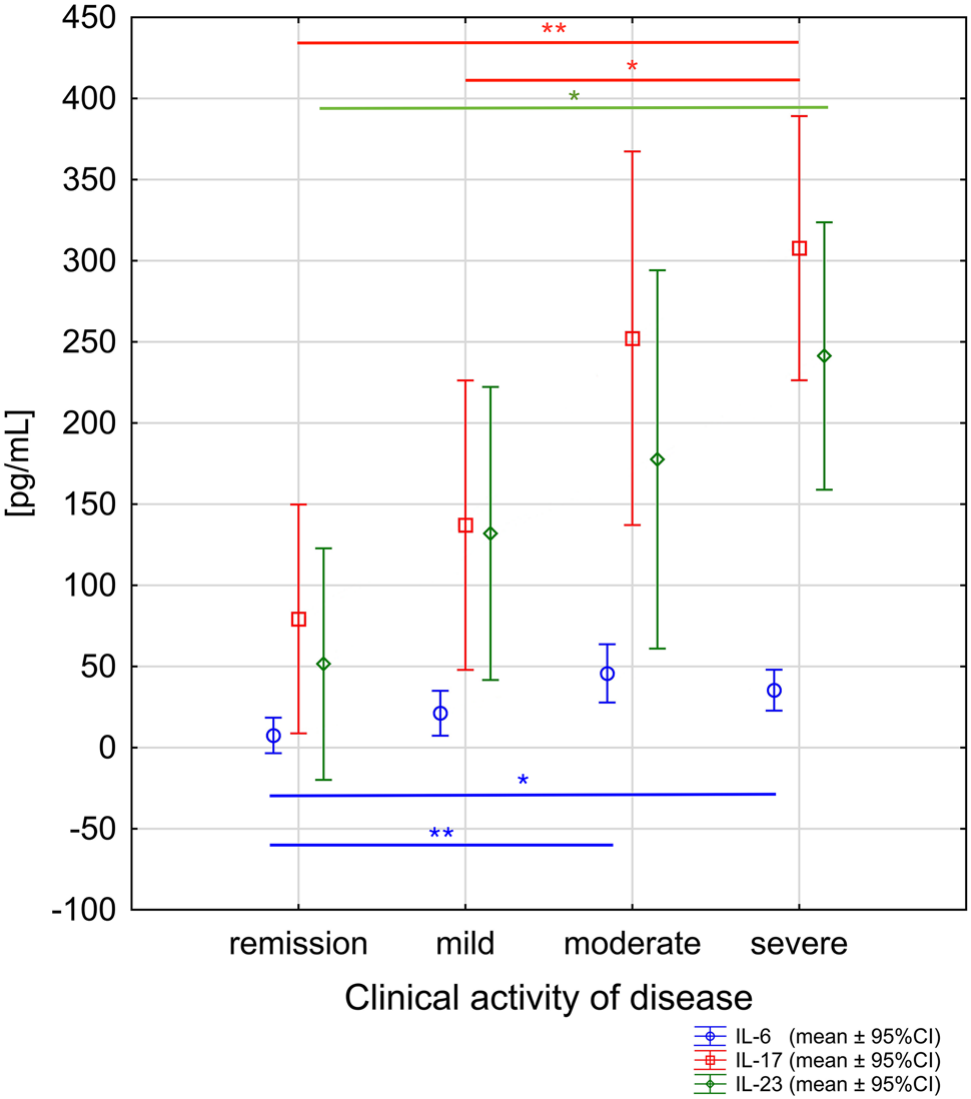
The relationships between serum levels of IL-6, IL-17, and IL-23 and clinical status of disease in inflammatory bowel diseases patients (IL-6: F3,18 = 6.74; *p* < 0.001; IL-17: F3,18 = 7.54; *p* < 0.001; IL-23: F3,18 = 4.65; *p* < 0.001). Data are expressed as mean ± 95% CI. Statistical significance from the one-way ANOVA followed by Tukey’s post hoc test Post hoc significant differences: IL-6: remission vs. moderate (*p* = 0.006); remission vs. severe (*p* = 0.013) IL-17: remission vs. severe (*p* = 0.002); mild vs. severe (*p* = 0.038) IL-23: remission vs. severe (*p* = 0.009)

According to the t test, the group of IBD patients with poor sleep, in comparison to group without sleep abnormalities had significantly higher levels of serum IL-6, IL-17 and IL-23 (*p* = 0.003, * t*  =  − 3.327, df = 20; * p*  = 0.004, * t*  =  − 3.311, df = 20; * p * < 0.001, * t* =  − 5.808, df = 20; respectively) (Figs. 3, 4 and  5). In our study the IBD patients with exacerbations of the intestinal lesions were characterized by significantly higher levels of serum IL-6, IL-17 and IL-23 (32.49 ± 19.57 vs. 7.46 ± 4.63 pg/mL, *p* = 0.002, * t* =  − 3.526, df = 20; 234.88 ± 127.09 vs. 79.28 ± 68.40 pg/mL, * p* = 0.005, *t* =  − 3.187, df = 20; 188.58 ± 132.33 vs. 51.47 ± 14.90 pg/mL, *p* = 0.006, *t * =  − 3.099, df = 20; respectively). In the one-way ANOVA analysis, the statistically significant differences were observed in serum levels of IL-6, IL-17 and IL-23 related to clinical state of IBD patients (IL-6: * F*_3,18_ = 6.74; *p* < 0.001; IL-17: * F*_3,18_ = 7.54; *p* < 0.001; IL-23: * F*_3,18_ = 4.65; *p* < 0.001) (Fig. 6).

Furthermore, there were statistically significant differences in the Pearson’s test linear correlations between CRP level and serum levels of IL-6, IL-17 and IL-23 (*r* = 0.649, *p* = 0.003; * r* = 0.498, *p* = 0.030; * r* = 0.759, *p* < 0.001; respectively). Furthermore, there was no statistically significant relationship between disease duration and serum levels of IL-6, IL-17 and IL-23 (r = 0.359, *p* = 0.207; r = 0.078, *p* = 0.183; r = 0.509 *p* = 0.063; respectively; Pearson’s test).

## Discussion

In this study, we confirmed a significant relationship between IBD and sleep disturbance. Specifically, 69.4% of all patients with clinically active disease included in the study manifested the significant quality of sleep abnormalities. Moreover, we have found that disease exacerbation is associated with significant sleep and serum proinflammatory cytokine profile abnormalities, in both CD and UC patients.

Physiologically, sleep is a recurring state characterized by altered, relatively inhibited sensory activity. In IBD patients, the influence of sleep disturbances on the course of the disease remains relatively unknown, with a very small number of recent investigations. As we have previously showed in recently published studies, there is a strong relationship between IBD activity and poor sleep conditions what may indicate that circadian rhythm disorders are a risk factor for IBD exacerbation [8, 9]. These observations have also been confirmed by the results of this study. ROC analysis confirms previous findings that PSQI higher than 6 indicates the exacerbation of IBD with high sensitivity and specificity [12].

Larussa et al. have highlighted that up to 35% of IBD patients, even in remission, showed a significant sleep disturbance, therefore suggesting that a state of low-grade chronic inflammation is still present affecting the circadian rhythm as well as the activity of the disease [14].

Here, a significantly higher levels of serum IL-6, IL-12, and IL-23 in IBD patients with poor sleep were observed. Furthermore, we also have found a significantly higher level of serum IL-6, IL-12 and IL-23 in IBD patients with clinically active disease. Until today, there are practically no studies on the relationship between sleep disturbances and the course of IBD and inflammatory cytokine profile. Proinflammatory cytokine profile among IBD patients is related with the overproduction of IL-1β, IL-2, IL-4, IL-10, IL-12, IL-18, and TNF [10–12]. The main proinflammatory cytokine responsible for the persistent inflammatory intestinal lesions seems to be TNF. Recently widely investigated other pathways in IBD suggested the crucial role of IL-23, IL-17A and interferon-γ (IFN-γ) in development of inflammatory in intestinal mucosa [12]. Hovhannisyan et al. observed the increased levels of IL-17A in serum and mucosa of CD patients, what underline the role of excessive activation Th17 lymphocyte pathway dependent on IL-23 in the pathogenesis of IBD [13, 14].

Sleep and the immune system have a bidirectional relationship. Immune response, like that caused by a viral infection, can affect sleep. At the same time, consistent sleep strengthens the immune system, allowing for balanced and effective immune function. Impaired quality of sleep, on the other hand, can deteriorate the immune system. Evidence indicates that in both the short and long term, sleep deprivation can lead to an immunological imbalance between pro- and anti-inflammatory cytokine levels and may increase the risk of exacerbation of immunological diseases [15, 16]. Relevant animal models confirmed the relationship between the increased levels of cytokines IL-1β, IL-6 and TNF, as well as CRP and sleep deprivation [17]. Importantly, these connection of proinflammatory cytokine profile and impaired quality of sleep have been confirmed in human studies [18, 19]. IL-1β and IL-6 enable transmigration of immunocompetent cells to sites of infection by the increased expression of adhesion factors on the surface of the endothelial cells [17]. Additionally, IL-6 is one of crucial factors in the acute phase response by stimulation of energy mobilization in muscle and fatty tissue that leads to increased body temperature and immunological response [20].

TNF is another cytokine that take a crucial role in development of systemic inflammation and belongs to group of cytokines that stimulate the acute phase response. It is released by activated CD4 + lymphocytes, natural killer (NK) cells, macrophages, and neurons. TNF is a potent paracrine and endocrine mediator of inflammatory and immune functions. It is also known to regulate growth and differentiation of a wide variety of cell types. TNF is selectively cytotoxic for many transformed cells, especially in combination with IFN-α, IL-1β and IL-6 producing cells [21].

Bidirectional interactions between the central nervous system and the immune system show that naturally occurring sleep acutely enhances the expression of immune markers implicated in immune defense and that inflammatory signals can promote sleep [22]. Poor sleep quality increase the activity of proinflammatory profile depended on excessive levels of IL-1β and IL-6 [23]. In the study by King et al. the short sleep measured by actigraphy was associated with an increased incidence of coronary artery calcification [24].

Consistent sleep has important effects on the GI tract physiological functions. The study by Hon et al. showed a threefold increase in the risk of bowel disorders in patients with self-reported insomnia compared to a control group in a Canadian population [25]. In the study conducted among the U.S. population the insomnia significantly decreases time to symptom generation and increases symptoms rating in patients with gastro-esophagus reflux disease (GERD) [26]. Another study showed that patients with GERD and irritable bowel syndrome (IBS) reported the higher rate of poorer sleep quality and incidence of nighttime awakening [26]. Sleep problems may also be related to side-effects of drugs or medication used in the treatment of IBD exacerbation such as steroids, mesalazine or aminosalicylic acid [27]. In our study no relationship between applied steroids in IBD patients and sleep abnormalities was observed.

Importantly, circadian rhythm abnormalities have been linked with altered intestinal permeability and IBD [28]. Evidence from studies in cell lines and in mice suggests that the expression of tight-junction proteins that constitute the intestinal epithelial barrier and determine permeability is under circadian control. Preuss et al. observed that chronic sleep deprivation rendered animals more susceptible to experimental colitis, increased the permeability of the intestinal epithelial barrier, and promoted a negative effect on colonic inflammation [29]. On the other hand, in the study by Zimmerman et al., the GERD symptoms and diarrhea in IBD and IBS patients impaired significantly the sleep quality [30]. It is noted, that 54% of pediatric IBD patients manifested moderate to severe sleep abnormalities [29]. Keefer et al. observed that IBD patients have significant objective (using polysomnography) and subjective (using PSQI survey) sleep deprivation in relation to the IBS group [31]. Various recent studies showed that sleep abnormalities affect the activity of inflammatory diseases. Graff et al. showed in a large cohort study, that up to 82% IBD patients with active disease defined by clinical symptoms and higher CRP levels manifested the poor sleep quality [32]. In the study by Ali et al., all IBD patients with clinically active disease reported the poor sleep quality assessed by PSQI score [31]. In a group of IBD patients with abnormal PSQI and clinical remission of bowel symptoms, 61% of patients had in colonoscopy mucosal or histological sings of inflammatory lesions. Further, the same group showed that the exacerbation relapse rate in IBD patients with poor sleep quality was 47% and 67% at 3 and 6 months, respectively, compared to 0% in patients with normal PSQI assessed at the same time intervals. Finally, the authors found that IBD patients in clinical remission with poor sleep quality have a high likelihood of subclinical disease activity [33].

The quality of sleep is related to the quality of life [34]. Sleep takes about 8 h each day. Poor sleep quality does not reduce strictly the quality of life by itself but can negatively affect other aspect of everyday life [35]. In patients with IBD, the impact of poorer quality of sleep on the overall quality of life has not been proven. However, it is influenced by many important elements, for example the underlying disease itself and its activity, and the quality of sleep may be one of them [35].

The potential limitations of our study should be considered. First, our cohort included mostly Caucasian adults, and the results may not be relevant to other ethnic groups. Second, our study included small sample size, indicating that the reported effect sizes might still be underestimated. Third, the study includes the lack of objective measurement of circadian rhythm abnormalities such as polysomnography. Furthermore, the study has not involved the controls without the IBD, and it is not possible to assess whether an association between cytokine profile and the quality of sleep is also present in healthy subjects. Finally, we used only the clinical status of IBD. Endoscopic evaluation and histopathological inflammation were not included. Currently, a prospective follow-up of included patients is ongoing to provide long-term follow-up data on the patients within our original cohort.

In conclusion, our study has proven a significant relationship between disease course in IBD patients and sleep abnormalities and the possible connection with the serum proinflammatory cytokine profile. The effectiveness of current IBD therapies is still unsatisfactory and studies for new approaches in the management of IBD patients are warranted for improvement of further treatment outcomes. Further studies on the relationship between circadian rhythm abnormalities and proinflammatory cytokine profile are needed to confirm a predisposition for the development of inflammatory intestinal lesions in IBD patients with sleep disturbances. This knowledge may allow the pharmacological and behavioral therapies of circadian rhythm abnormalities to become new significant targets in the management of IBD patients.

## Abbreviations

IBD: Inflammatory bowel disease
CDAI: Crohn’s disease activity index
CD: Crohn’s disease
UC: Ulcerative colitis
PSQI: Pittsburgh quality sleep index
IL: Interleukin
TNF: Tumor necrosis factor
IFN-α: Interferon-α
IFN-γ: Interferon-γ
NREM: Non-rapid eye movement
REM: Rapid eye movement
SWS: Slow-wave sleep
SD: Standard deviation
ECCO: European Crohn’s and Colitis Organization
CRP: C-reactive protein
ELISA: Enzyme-linked immune sorbent assay
ROC: Receiver operating characteristic
CI: Confidence interval
PPV: Positive predictive values
NPV: Negative predictive values
GERD: Gastro-esophagus reflux disease
IBS: Irritable bowel syndrome
NK: Natural killer
